# Chicago Dental Society

**Published:** 1889-03

**Authors:** Louis Ottofy

**Affiliations:** Corresponding Secretary.


CHICAGO DENTAL SOCIETY.—At the regular meeting of this Society, held Tuesday evening, March 5th, 1889, the following resolution was adopted:

*Resolved*, That it is the sentiment of this Society that it would be of interest to the members of the dental profession to become members of the Dental Protective Association of the United States.

LOUIS OTTOFY,
Corresponding Secretary.

